# Multiple roles of motor imagery during action observation

**DOI:** 10.3389/fnhum.2013.00807

**Published:** 2013-11-25

**Authors:** Stefan Vogt, Franck Di Rienzo, Christian Collet, Alan Collins, Aymeric Guillot

**Affiliations:** ^1^Department of Psychology, Lancaster UniversityLancaster, UK; ^2^Centre de Recherche et d'Innovation sur le Sport, équipe Performance Motrice, Mentale et du Matériel, Université de Lyon, Université Claude Bernard Lyon 1Villeurbanne, France; ^3^Institut Universitaire de FranceParis, France

**Keywords:** motor simulation, mirror neurons, joint action, observational practice, mental practice, video therapy, occupational therapy, motor rehabilitation

## Abstract

Over the last 20 years, the topics of action observation (AO) and motor imagery (MI) have been largely studied in isolation from each other, despite the early integrative account by Jeannerod (1994, 2001). Recent neuroimaging studies demonstrate enhanced cortical activity when AO and MI are performed concurrently (“AO+MI”), compared to either AO or MI performed in isolation. These results indicate the potentially beneficial effects of AO+MI, and they also demonstrate that the underlying neurocognitive processes are partly shared. We separately review the evidence for MI and AO as forms of motor simulation, and present two quantitative literature analyses that indeed indicate rather little overlap between the two bodies of research. We then propose a spectrum of concurrent AO+MI states, from congruent AO+MI where the contents of AO and MI widely overlap, over coordinative AO+MI, where observed and imagined action are different but can be coordinated with each other, to cases of conflicting AO+MI. We believe that an integrative account of AO and MI is theoretically attractive, that it should generate novel experimental approaches, and that it can also stimulate a wide range of applications in sport, occupational therapy, and neurorehabilitation.

## Introduction

In this paper we contribute to the emerging integration of research on action observation (AO) and motor imagery (MI). We outline a coherent account of both forms of action representation, which have been typically studied in their own right and by different scientific communities and sub-communities (Moran et al., 2012). Our first point is not new: observers can engage in AO and MI simultaneously (“AO+MI”), and doing so does not take particular skill. Such integrated AO+MI appears to be more pervasive than either form of action representation alone. Our second and main point is that the contents of such simultaneous AO+MI need not coincide. We propose that there is a spectrum from fully congruent AO+MI, where the observer imagines performing the observed action, perhaps through periods of partial occlusion from sight and enriched by the imagined kinesthetic sensations that would arise during one's own motor execution, through to scenarios where the contents of AO and MI conflict, that is, where co-representation of two different actions is difficult or impossible to sustain and where markers of representational depth indicate competition. Lying between the extremes of congruent AO+MI and incongruent, conflicting contents of AO and MI is the co-representation of two different actions that can be coordinated in some manner. For example, in combat sports, I might watch a video recording of a future opponent whilst simultaneously imagining myself performing specific technical attacks or defense movements against that opponent. We believe that this proposed spectrum from congruent over coordinative to conflicting AO+MI states will motivate researchers to probe the two component processes, as well as their interaction, in greater depth than previously undertaken. At the same time, we can see tremendous opportunities for examining the application of various forms of concurrent AO+MI in motor learning and neurorehabilitation.

Our article is organized as follows: In the first section, we turn to the field of motor rehabilitation, where a number of research groups have already made a research-based case for combining AO and MI. Both forms of action representation have been proposed as promising adjunct treatments to conventional physiotherapy, but an integrated approach to treatment is still largely absent. We review recent neuroimaging studies which underpin the proposal of integrating AO and MI in motor rehabilitation and briefly point to future opportunities and open questions. In section “Action observation and motor imagery—a continuum,” we outline an integrative account of AO and MI as motor simulation, inspired by the early contribution by Shepard (1984). In section “Motor imagery as motor simulation,” we review the evidence, from both behavioral and neuroimaging studies, for MI as a prototypical form of action simulation, as originally proposed by Jeannerod (2001, 2006). In section “Research on action observation and motor imagery,” we turn to research on AO, which has generated comparable evidence for motor simulation during AO. We then present evidence from two quantitative literature analyses for the rather scarce overlap between research on MI and on AO, and we discuss the links that have previously been made between the two forms of action representation. In section “Multiple roles of motor imagery during action observation,” we then describe the full spectrum of AO+MI states as outlined above, along with possible training applications. On a theoretical level, we propose a distinction between a default mode of action simulation during AO and a more specific AO+MI state where the observer actively maps the observed action onto her/his own body schema via engaging in MI.

## A case for motor imagery during action observation

In motor rehabilitation, both MI and AO have been proposed as adjunct treatments to conventional physiotherapy (e.g., Mulder, 2007; Garrison et al., 2010). The available clinical studies demonstrate varied success of both MI (e.g., Crosbie et al., 2004; Dijkerman et al., 2004; Page et al., 2007; Ietswaart et al., 2011; for review see Braun et al., 2013; Malouin et al., 2013, this issue) and AO therapy (Ertelt et al., 2007; Celnik et al., 2008; Ewan et al., 2010; Franceschini et al., 2010; Cowles et al., 2013; for review see Gatti et al., 2013), and a multi-center study on AO therapy is currently underway (Ertelt et al., 2012). Typically only one form of treatment, either MI or AO, has been used as an intervention [for an exception, see Ietswaart et al. (2011)], possibly with the conclusiveness of the clinical trial in mind. However, such a “purist” approach ignores the possible benefits of a multimodal motor simulation training with AO and MI as integrated components. For example, in some of our electrophysiological and neuroimaging studies, we have deliberately combined instructions for AO and MI in the practice phases (e.g., Wehner et al., 1984; Higuchi et al., 2012), with the aim of maximizing the benefits of non-physical forms of practice, even though doing so precluded specific conclusions about the effects of pure AO vs. pure MI vs. combined AO+MI.

Fortunately, a number of recent neuroimaging studies have directly contrasted these conditions using healthy participants. Filimon et al. (2007) compared activations during AO, MI (visuo-motor imagery without visual input), and during execution of reaching movements, and they found differences between AO and MI only in occipital (visual motion) regions. Furthermore, motor execution induced stronger activations than either AO or MI in a number of execution-related areas, including primary sensorimotor areas, posterior parietal cortex, and dorsal premotor cortex [see also Vogt et al. (2007), for similar results during observation, preparation, and motor execution of a complex grasping task]. In no less than four recent neuroimaging studies, passive observation was contrasted with combined AO+MI, where the instructions required participants to imagine performing the displayed movement from a first-person perspective (Macuga and Frey, 2012; Nedelko et al., 2012; Berends et al., 2013; Villiger et al., 2013). Nedelko et al. (2012) designed their conditions to match their video therapy sessions with stroke patients and included videos of simple and multiphasic hand-object interactions, as well as pantomimed actions. Combined AO+MI induced stronger activations than passive AO in inferior parietal cortex, supplementary motor area (SMA), inferior frontal gyrus (IFG), caudate nucleus, and the cerebellum. Macuga and Frey (2012) contrasted passive AO, AO+MI, and AO plus imitative execution of bimanual finger sequences. In line with the results of Filimon et al. (2007), imitative execution induced stronger activations in a number of execution-related areas. More importantly, compared to passive AO, combined AO+MI increased activations in the pre-SMA and left IFG, as well as cingulate cortex and anterior insula. Further, a manipulation of visual perspective of the observed action (1st- vs. 3rd-person) only produced differences in occipital regions, which the authors attributed to differential stimulation of the lower and upper visual fields in their paradigm. In the fMRI study by Villiger et al. (2013), essentially the same three conditions as in Macuga and Frey (2012) were compared for first-person displays of a kicking action. MI during AO resulted in enhanced activations relative to AO alone in bilateral ventral premotor cortex, left inferior parietal cortex, and left insula. Further, a conjunction analysis of AO+MI and AO plus imitative execution showed a substantial overlap between the related activations in motor cortical areas, indicating that large parts of the motor execution network can be activated during AO+MI. Finally, Berends et al. (2013) demonstrated that the differences between combined AO+MI and AO alone can also be demonstrated using EEG. The authors found substantially larger desynchronizations during AO+MI, where participants observed movie clips of repeated pincer grips.

These studies highlight two important points. First, they strengthen the evidence for a considerable overlap between AO, AO+MI, and visually guided motor execution, in that all three forms of action representation involve a bilateral network within posterior parietal and frontal premotor cortex, also known as the “AO network” [see also meta-analysis by Caspers et al. (2010), and section “Research on action observation and motor imagery” below]. Second, AO+MI induced stronger activations in certain regions of this network than observation alone. On this basis, all four research teams recommended the use of combined AO+MI procedures in neurorehabilitation.

It should be noted, however, that stronger activations are not always “better,” and that differential activations whilst engaging in action representation instructions do not allow direct inferences about the possible effects on skill learning. The study by Higuchi et al. (2012) illustrates this point: Participants were scanned during observational practice (involving combined AO+MI) and, in separate scanning sessions, during imitative execution of manual actions (guitar chords) that had previously been practiced either via AO combined with MI, or via physical practice. As expected, when scanned during AO, a common network involving posterior parietal and premotor regions was found activated, with only minor differences between the two forms of practice (see also Cross et al., 2009). During imitative execution, the results were strikingly different: Chords that had been observationally practiced induced substantially stronger activations during imitative execution than the physically practiced chords. Given that the behavioral data indicated smaller practice effects for the observationally practiced actions than for the physically practiced actions, and given the general trend for the cortical activations to reduce with increasing practice (“neural efficiency,” Kelly and Garavan, 2005; Babiloni et al., 2009, 2010), these results indicated a lack of execution-related resources in observationally practiced actions. Importantly, however, when compared with non-practiced actions the observationally practiced actions also exhibited neural efficiency effects. Thus, whilst the study by Higuchi et al. (2012) reminds us that we cannot normally expect non-physical forms of practice to produce the same results as physical practice, it also indicates that AO+MI procedures can have substantial benefits. In future, it would be desirable that imaging studies contrast the practice effects of different forms of action representation, such as pure AO, pure MI, and AO+MI.

Taken together, the studies reviewed in this section not only illustrate the feasibility of simultaneous AO+MI instructions, they also demonstrate the immediate facilitatory effects of combining AO with MI, as well as longer-term positive effects on motor learning (sensu neural efficiency). Whilst further clinical trials are needed to confirm these effects in neurorehabilitation (Ertelt et al., 2012), the above studies clearly indicate that AO and MI training should not be seen as mutually exclusive means of treatment, but that their combined and simultaneous usage can be highly recommended. This conclusion will hopefully empower physiotherapists to develop and apply a wide range of tasks to help patients to (re-)engage in motor simulation processes. Many open questions remain at present, such as the suitability of specific subforms of motor simulation training for particular patient groups, the most suitable design of video therapy materials, and which perspective and modality instructions might be most appropriate.

## Action observation and motor imagery—a continuum

**Figure 1:**
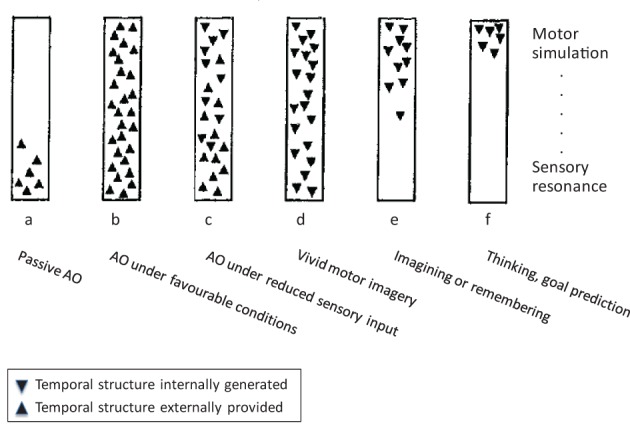
**The continuum between action observation and motor imagery, modified from Shepard (1984), for details see text**. Note: Whereas Shepard's scheme encompassed a wider range of cognitive processes, we focus on AO and MI. In addition, we have replaced Shepard's ordinate of a “hierarchy of resonant modes” (from most concrete and sensory at the bottom to most abstract and conceptual at the top) by a sensory-motor axis.

Roger Shepard once caricatured “*perception as externally guided hallucination, and dreaming and hallucination as internally simulated perception*” (Shepard, 1984, p. 436). Similarly, we see AO as externally guided motor simulation, and MI as internally simulated execution. The idea that motor simulation might underlie both AO and MI was originally proposed by Jeannerod (1994, 2001, 2006), and more recently motor simulation, along with prediction as its most prominent cognitive function, has become a commonly accepted framework for a wide range of cognitive domains (Grush, 2004; Kilner et al., 2007; Bubic et al., 2010; Pezzulo et al., 2013). Before we turn to motor simulation in AO and MI in greater detail, we wish to illustrate the possible relationships between them by means of Figure 1, which is adapted from Shepard (1984) but reframed for the present purposes. Both schemata aim to distinguish different “*externally and internally instigated representational processes*” (ibid., p. 435).

### Action observation

With reference to Figure 1, the most frequently studied case of AO is (b), which represents observation of another person's action under favorable viewing conditions. The schema firstly suggests that this proceeds (rapidly) from sensory processing of the observed action to motor simulation processes (e.g., Eskenazi et al., 2009; Zentgraf et al., 2011; for discussion, see Kilner, 2011). Second, the orientation of the triangles indicates the externally driven character of sensory and motor processes in this case. This implies that motor simulation processes are not only initiated by sensory events but that they can also unfold in close coupling to external, temporally extended events such as observed actions. Rectangle (a) in Figure 1 indicates that motor simulation processes are not mandatory in AO. For instance, in the domain of speech perception, Scott et al. (2009) concluded that motor simulation processes are more heavily involved under impoverished stimulation (e.g., distorted speech) so that (a) would represent the normal case in this domain. Whereas motor involvement in AO is a more typical finding than in speech perception, the latter at least illustrates the possibility of AO without the involvement of simulation processes. For instance, drawing on the study by Buccino et al. (2004), Rizzolatti and Sinigaglia (2010) conclude that “*these data indicate that the recognition of the motor behavior of others can rely on the mere processing of its visual aspects*” (ibid., p. 270; see also Gallese et al., 2011). Finally, rectangle (c) indicates that motor simulation during AO does not rely on continuous concurrent sensory guidance but can also proceed under reduced visual input, such as transient occlusion.

### Motor imagery^1^

Rectangles (d) to (f) represent wholly internally driven motor simulation. We propose that vivid MI can invoke the full spectrum of sensory and motor representation (d), whereas less vivid instances of MI, remembering, and goal prediction (e, f) might lack specific sensory features but still originate in motor simulation. In line with this, MI is commonly defined as the mental simulation of one's own performance without any associated overt movement (Jeannerod, 1994). It involves a subset of the neurocognitive preparatory and “real-time” processes of motor execution. More specifically, the preparatory phase of both motor execution and MI (Vogt, 1994) typically includes the anticipation of distal and proximal action effects (e.g., Ziessler et al., 2012) along with, for example, an action-oriented processing of object properties (Milner and Goodale, 2008). The real-time processes during both execution and MI further involve a “sense of effort” (James, 1890), agency (Frith, 2010, 2013), the experienced or simulated kinesthetic and other sensory input, and related monitoring operations (Shallice, 2004). One main difference between actual and imagined movement is that during the latter, motor commands are inhibited throughout the motor system to prevent overt execution (Guillot et al., 2012a). Practically, inhibition during MI may be a functional process resulting from the specific contribution of neural sites usually dedicated to overt motor processing. From a multifactorial viewpoint, motor inhibition might involve both cerebral and spinal mechanisms, and three possible routes for motor command inhibition during MI have been proposed in the literature (Guillot et al., 2012a).

In summary, Figure 1 introduces the notion that AO and MI can involve a similar range of sensory and motor representational processes that constitute a continuous descriptive framework, where the two principal dimensions are the external vs. internal origin, and the emphasis on sensory resonance vs. motor simulation. AO and MI differ in that AO can involve motor simulation to varying degrees, and that it can rely on both external and (in part) internal guidance, whereas MI proceeds by definition in the absence of external guidance, and it can vary in the concreteness of sensory representation. These proposals will be elaborated in the next two sections.

## Motor imagery as motor simulation

The ability to imagine is one of the most remarkable capacities of the mind to simulate sensations, actions and other types of experience. Morris et al. (2005, p.19) defined imagery as “*the creation or re-creation of an experience generated from memorial information involving quasi-sensorial, quasi-perceptual and quasi-affective characteristics, that is under the volitional control of the imagery, and which may occur in the absence of the real stimulus antecedents normally associated with the actual experience.*” Within this general definition, the process of imagining motor execution is known as MI. MI is a multimodal construct based on distinct sensory modalities, and there is compelling evidence that different imagery modalities and imagery types can be performed, with visual and kinesthetic imagery being probably the most frequently reported. Diary imagery studies have shown that about two thirds of our mental images are visual in nature (Moran, 2002). During internal visual imagery (first-person perspective), people visualize the action as it would happen in real-life and see images as if through their own eyes. During external visual imagery (third-person perspective), people imagine, like spectators, the action that somebody is performing, regardless of the agency of that movement (i.e., whether they “see” themselves or others performing it). By contrast, kinesthetic imagery involves the sensations of how it feels to perform an action, including the force and effort perceived during movement, hence suggesting the body as a generator of forces (Jeannerod, 1994). Practically, these definitions suggest that MI is *the* prototypical form of motor simulation (Jeannerod, 2001, 2006). While one can consider that pure visual imagery—i.e., without engaging in motor simulation—is possible (e.g., think about consequences of different actions abstractly), MI requires a motor strategy in almost all situations. In his motor simulation theory, Jeannerod (2006, p. 130) postulated that represented actions might involve a large subset of the mechanisms that usually participate in the various stages of action generation, including motor execution.

A significant number of experimental and neuroimaging studies support the proposal that MI involves motor simulation. First evidence comes from mental chronometry work, where researchers compared the time taken to imagine a movement with that needed to actually perform it (for review, see Guillot et al., 2012b). Since the pioneering contribution on this topic by Decety et al. (1989), a handful of experimental studies have shown that participants take the same time to achieve both physical and mental tasks. This is known as the principle of temporal congruence, which is based on motor prediction of the temporal features of the movement to be imagined. While there are several influencing factors likely to affect imagery times (Guillot and Collet, 2005), mental chronometry data strongly support that participants engage in motor simulation of the actual movement during MI by predicting as accurately as possible the temporal features of the corresponding action. A second line of evidence derives from recording the autonomic nervous system activity during MI. In their recent review, Collet et al. (2013) conclude that engaging in MI requires motor planning and programming operations, and anticipating the possible consequences of an action, such brain operations being accompanied by a set of physiological responses which can be recorded at the level of peripheral effectors. There is now ample evidence that MI and physical practice of the same movement elicit similar autonomic nervous system responses (e.g., Decety et al., 1991; Wuyam et al., 1995; Roure et al., 1999), and that imagery ability and efficacy can even be objectified and evaluated through autonomic responses (Collet et al., 2011).

Neuroimaging experiments also support the contention that MI involves motor simulation. Understanding the neural correlates of goal-directed action, whether executed or imagined, and exploring the neural underpinnings of different kinds of MI, has been an important purpose of cognitive brain research for the last three decades (for reviews, see Jeannerod, 1994; Grèzes and Decety, 2001; Nyberg et al., 2006; Munzert et al., 2009; Hétu et al., 2013). Briefly, studies have demonstrated that MI engages motor systems, and that the cerebral plasticity resulting from actual practice also occurred as a result of MI. These findings help to explain why MI can improve actual performance, and further contribute to motor memory consolidation. Of specific interest is the strong overlap between the neural networks mediating MI and the corresponding substrates activated during physical practice. Interestingly, Ehrsson et al. (2003) found that MI of hand, foot and tongue movements specifically activated the corresponding hand, foot and tongue sections of the primary motor cortex, hence suggesting specific motor simulation processes during MI. A similar conclusion can be drawn from studies comparing the neural networks activated during visual and kinesthetic imagery (Solodkin et al., 2004; Guillot et al., 2009), as motor systems were found to be more active during kinesthetic imagery, which is closer to actual practice and requires considering the body as a generator of forces to simulate the movement. Finally, a seminal clinical study in a patient with bilateral parietal lesions showed a complete unawareness of movement execution during imagery, where the patient exhibited hand movements during MI of the same body segments while explicitly denying that they occurred (Schwoebel et al., 2002). In other words, this patient engaged in complete motor simulation but failed to inhibit the motor consequences of MI which usually preclude actual movement.

A last line of (indirect) evidence of motor simulation during MI comes from experimental studies showing practice and instantaneous priming effects. Many studies of mental practice effects have demonstrated the efficacy of MI for improving motor performance and consolidation (for reviews, see Feltz and Landers, 1983; Driskell et al., 1994; Weinberg, 2008; Schuster et al., 2011). Such simulation of movements may engage relevant motor-related areas and might further build associations among processes implemented in different areas, hence facilitating subsequent motor execution (Jeannerod, 2001; Kosslyn, 2010). Recently, Ramsey et al. (2010) further demonstrated that imagining an action that was different to the to-be-performed action interfered with action execution. This finding shows that MI is likely to prime the motor system to produce the action, hence supporting that MI involves motor simulation processes (see also Vogt, 1995, 1996).

## Research on action observation and motor imagery

While the general topic of mental imagery, if not MI itself, is one of the oldest areas of inquiry in psychology (Galton, 1883; James, 1890; Sully, 1892; Titchener, 1909), by contrast, “action observation” as a phrase seems not to have become prominent in the psychological literature until the 1990s, following a series of much-cited papers on mirror neurons and their properties (Di Pellegrino et al., 1992; Gallese et al., 1996; Rizzolatti et al., 1996, see Rizzolatti and Fabbri-Destro, 2010). Of course, this is not to say that observation of action was ignored by psychological research prior to this: it clearly was not. However, AO was given a new, or renewed, significance by the discovery of mirror neurons and developments in the understanding of perception-action links. Whereas the computational stages in MI, from the intention to act to real-time imagery, are most likely highly similar to those in non-imagined actions, the notion of direct links between AO and the motor system is less intuitive, and only over the last two decades, theorizing in neuroscience and psychology has fully embraced the latter idea. We now briefly recapitulate these developments, with a view on the commonalities and differences between AO and MI.

The discovery of mirror neurons in the macaque monkey was made in the context of *motor* neuroscience (Rizzolatti and Fabbri-Destro, 2010). The original findings opened up the possibility of establishing action understanding as a new, *cognitive* function of the motor system, and this pursuit has been a strong driver of the related research from its very beginning (Rizzolatti and Sinigaglia, 2010). Furthermore, once the existence of mirror neurons was established, for experimental scientists in various disciplines the study of AO and related imitative phenomena promised to illuminate intuitively appealing psychological topics such as empathy and theory of mind (but see Frith and Frith, 2006, 2012; Van Overwalle and Baetens, 2009). This prompted an impressive research effort into potentially similar mirror mechanisms in the human brain (Rizzolatti and Sinigaglia, 2010). Whereas the number of human brain regions with mirror properties and their exact functions is still under debate (e.g., Rizzolatti, 2005; Gallese et al., 2011), a large number of neuroimaging studies have demonstrated that motor cortical structures in the ventral and dorsal premotor cortex and in the adjacent caudal sector of the IFG are typically activated during AO, together with visual temporal and posterior parietal regions (Caspers et al., 2010), as well as somatosensory cortex (Keysers et al., 2010). Together these regions are also known as the “AO network.”

As we have already noted in section “A case for motor imagery during action observation,” a fairly large overlap of activations was found in the few studies that have directly compared AO and MI, possibly indicating that basic motor simulation processes are shared between MI and AO. Kilner (2011) recently proposed a two-process account of AO, where the initial action recognition (via a ventral temporo-frontal pathway) is segregated from motor simulation (via the parieto-frontal mirror circuit). This proposal does not preclude the rapid and simultaneous operation of the two processes, and it helps to clarify our present focus on the second process in Kilner's framework, motor simulation (Pezzulo et al., 2013). Motor simulation, in the sense of an internal, on-line representation of the observed action, is particularly useful when the observer needs to predict a certain temporal landmark (e.g., object release) of the observed action for purposes such as attuning one's own action toward this landmark or synchronizing one's own action with the observed action. A particularly impressive demonstration of the close coupling between observed actions and the observer's motor system was provided by Borroni et al. (2005), who showed that the excitability of the motor system exhibited a cyclical time course that closely matched that of an observed rhythmical action. In fact, such motor simulation, or “motor resonance” (Rizzolatti et al., 2002) is so universally useful that it might be described as a default mode of visuo-motor processing during AO. Finally, Schubotz (2007) and Bubic et al. (2010) have generalized this form of action prediction beyond actions that are in the behavioral repertoire of the observer and demonstrated that the premotor cortex is also involved in the prediction of non-biological events and event sequences. In summary, the available neuroimaging studies clearly support the notion of motor simulation as a default mode of AO, which can subserve a variety of functions.

In psychological research, the seminal reaction time studies by Brass et al. (2000) and Stürmer et al. (2000), both conducted in W. Prinz' perception-action group at the Max-Planck Institute for Psychological Research, motivated a large set of studies on visuomotor priming or “automatic imitation” (for reviews, see Vogt and Thomaschke, 2007; Heyes, 2011). Basically, these studies show that observed actions can bias the speed and accuracy in which similar actions are performed, and they thus provide behavioral evidence for direct links between AO and motor planning. A central feature of these studies is that the observed actions are normally irrelevant for the observer's own action planning, which strengthens the notion of low-level, automatic perception-action links. A second feature of this line of research was its focus on static depictions of actions [e.g., the prototypical lifted index finger of Brass et al. (2000)], although more recently automatic imitation effects have also been documented for temporally extended actions, such as everyday rhythmical actions (Eaves et al., 2012).

As already pointed out in section “Action observation and motor imagery—a continuum,” a key difference between AO and MI is their external vs. internal origin. AO involves the sensory processing and attunement to the partly unpredictable action “out there,” whereas these processes are by definition not part of MI. AO thus includes a wider range of neurocognitive processes than MI, particularly action recognition and intention understanding (Rizzolatti and Sinigaglia, 2010), action prediction (Springer et al., 2013), and collaborative action (either imitative or complementary, joint action, Bekkering et al., 2009). In the present paper, we focus on an instance of AO which exhibits the greatest similarity to MI, namely the repeated observation of largely predictable action displays, such as the repeated observation of object grasping as used in motor rehabilitation (Ertelt et al., 2007; Nedelko et al., 2012). As described above, a large number of both neuroimaging and behavioral studies confirm the involvement of motor processes in this form of AO. Notwithstanding the considerable overlap between AO and MI in this respect, AO surely encompasses additional neurocognitive processes.

This brief review of neuroimaging and behavioral research on AO reinforces the idea that, until now, the two bodies of research have not been particularly interested in the possible commonalities between AO and MI. Both neuroscientists and psychologists were (understandably) attracted by the opportunity to manipulate visual displays, rather than MI instructions, and to demonstrate visuomotor priming effects independently of the observer's task instructions. Likewise, researchers working on MI have rarely explored the virtues of using task-irrelevant displays, given that participants can be directly instructed to engage in MI tasks. That is, as already noted in the Introduction, until now the processes of AO and MI have, with a few notable exceptions, been considered separately and investigated by different groups of scientist^2^ (for different sub-communities within research on MI, see Moran et al., 2012). In the remainder of this section, we present two quantitative literature analyses which tentatively support this claim, and then turn to previous points of contact and attempts of integration between the two research fields.

### Highlights of the AO / MI literature

One way to demonstrate a lack of overlap between research on AO and MI is to revisit the related meta-analyses. Interestingly, the first such meta-analysis (Grèzes and Decety, 2001) encompassed motor execution and MI, as well as AO. The more recent meta-analyses, however, are either focused on AO (Caspers et al., 2010, on AO and imitation; Van Overwalle and Baetens, 2009; Grosbras et al., 2012, on AO and metalizing) or on MI (Hétu et al., 2012, 2013), but not on both. Despite the wholly legitimate, narrower focus of these recent meta-analyses, overlap between the underlying individual studies would still be conceivable. However, a comparison between Caspers et al.'s (2010) and Hétu et al.'s(2013) meta-analyses shows surprisingly little overlap: Of the 87 studies on cortical activations during AO and imitation that were included in the meta-analysis by Caspers et al. (2010), just four are also cited in Hétu et al.'s(2013) meta-analysis on the neural correlates of MI. Even allowing for different papers being reviewed in order to justify a novel contribution, this number is small. And of the 335 papers cited by the two articles together, just 18 are cited by both. This offers at least a prima facie case for claiming that the two research areas have not overlapped to the extent one might have expected.

**Figure 2:**
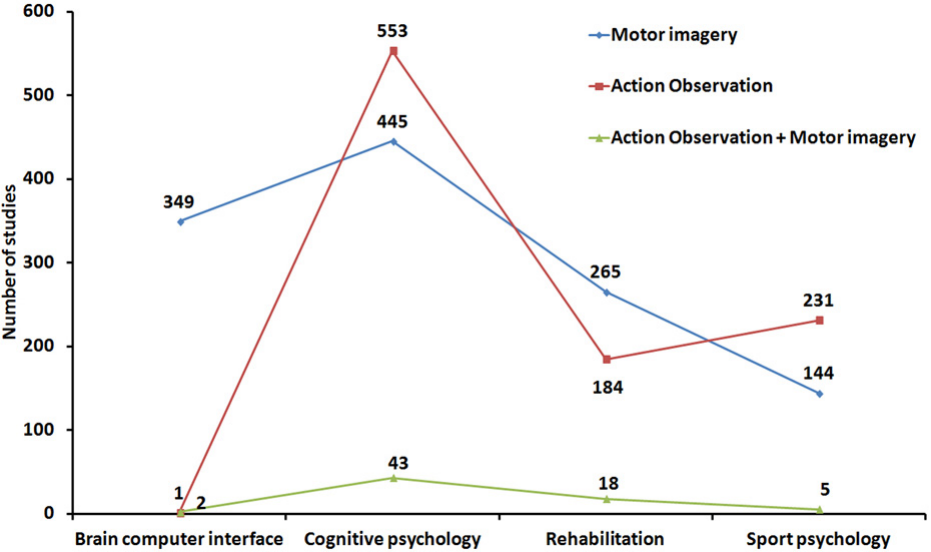
**Percentage of AO, MI, and AO+MI studies in the four pre-determined research areas**.

Looking more widely at the impressive literature dealing with these two research areas confirms that MI and AO have been largely studied in relative isolation from each other. For instance, over the last 20 years, both MI and AO have been shown to contribute to improve motor performance and facilitate motor recovery, but few researchers have investigated whether MI and AO might be combined or considered in a common framework. We performed a literature search from the PubMed database by selecting indexed articles related to (*i*) motor/movement/action imagery and (*ii*) motor/movement/AO and action imitation. A large sample of 2172 references (including review papers) met the topical inclusion criteria (*note that a substantial number of sport-related references do not appear in the Pubmed database and were therefore not considered in this illustrative overview of MI and AO research areas. This may explain the unexpectedly small number of MI studies in Sport psychology*). 1203 articles investigated MI while 969 focused on AO. Each reference was then categorized as a study on either brain computer interface (BCI), cognitive psychology, rehabilitation, or sport psychology (Figure 2). The lack of AO research in BCI is basically expected and trivial. A larger number of AO studies than of MI studies was found in cognitive psychology, while the reverse was true in rehabilitation, which makes sense. The higher number of AO studies in sport psychology is more surprising, but the list of references retrieved from our chosen database is not exhaustive in this specific area.

The most important outcome of this analysis is that only 68 articles (3.1% of the sample) considered both AO and MI concurrently (including 14 review papers—2 in Cognitive psychology, 2 in Sport psychology and 10 in Rehabilitation). The most famous integrated accounts of AO and MI can be found in seminal theoretical papers (Shepard, 1984; Jeannerod, 1994, 2001, 2006; Annett, 1996). These authors specifically considered both the prescriptive nature and the neural models of action representations. Holmes and Calmels (2008, 2011) later contrasted the definitions and benefits of AO and MI. However, none of these important contributions was really designed to consider the possible role of MI during AO, either when AO and MI are congruent or incongruent. Few neuroimaging studies have considered both AO and MI, and the neural underpinnings of AO and MI were largely studied in isolation until more recently, when more detailed overviews of the substrates of action simulation have been provided (Munzert et al., 2009; Lorey et al., 2013a,b). Only a handful of researchers even considered concurrent AO+MI (see section “A case for motor imagery during action observation”). The advent of transcranial magnetic stimulation and the study of corticospinal excitability increased the number of studies contrasting and/or combining AO and MI (Clark et al., 2004; Leonard and Tremblay, 2007; Tremblay et al., 2008; Conson et al., 2009; Liepert and Neveling, 2009; Sakamoto et al., 2009; Battaglia et al., 2011; Feurra et al., 2011; Loporto et al., 2011; Bianco et al., 2012; Tsukazaki et al., 2012). Furthermore, researchers investigating BCI systems now consider the impact of both AO and MI on the modulation of brain rhythms (e.g., Neuper et al., 2009). Finally, some experimental studies in the field of sport psychology (Lejeune et al., 1994), cognitive psychology (Vogt, 1996; Conson et al., 2009; Ramsey et al., 2010; McCormick et al., 2012; Williams et al., 2012), as well as review studies in the field of motor rehabilitation (Mulder, 2007; Johansson, 2012) have investigated the respective effects of MI and AO and whether AO primes or improves MI [for a review on learning effects, see also Gatti et al. (2013)].

Basically, most of the studies mentioned above contrasted AO and MI, only very few considered concurrent AO+MI, and until recently, none had considered coordinative or conflicting AO+MI (see below). Curiously, several researchers opposed AO and MI in order to find which technique is likely to be optimal in enhancing performance. For instance, Holmes and Calmels (2008, 2011) stated that observation can provide some solutions to the problems identified in the use of imagery (e.g., image generation and maintenance, behavioral agency, control of visual perspective, and viewing angle) and offers a more ecologically valid environment for addressing many sporting tasks. Whilst this is probably sound in some circumstances and their examples are well-illustrated, it is unclear whether or not observation as conceptualized by Holmes and Calmels is accompanied by the mental representation of the corresponding action sensu MI. Another example comes from instructions delivered in some MI experiments where researchers have drawn conclusions about MI use when the participants were actually asked to engage in combined AO and MI (Macuga and Frey, 2012). All combinations of AO+MI procedures will now be detailed in section “Multiple roles of motor imagery during action observation,” in order to provide a better overview of the possible associations and differences between AO and MI.

## Multiple roles of motor imagery during action observation

**Figure 3:**
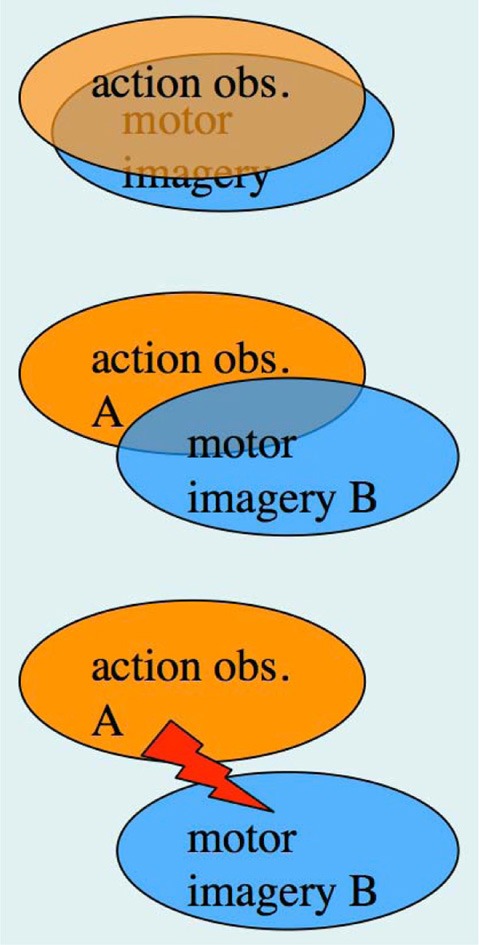
**The spectrum of concurrent AO+MI states. Top panel:** congruent AO+MI; **Center panel:** coordinative AO+MI, where two different actions A and B are co-represented in some form; **Bottom panel:** conflicting AO+MI (see text for details).

In the previous section, we have pointed out that research on AO and MI has been carried out, to a large extent, by different research groups, despite the fact that integrative accounts of AO and MI as sub-forms of action representation, or action simulation, have been available for quite some time (Shepard, 1984; Jeannerod, 1994, 2001, 2006). The possibility of concurrent AO+MI states, however, was not featured in the above accounts. In section “A case for motor imagery during action observation” we have already made a case for concurrent AO+MI, based on the recent neuroimaging studies by Macuga and Frey (2012), Nedelko et al. (2012), and Berends et al. (2013). We now explore the full spectrum of AO+MI states (Figure 3), and begin with perhaps the most practically relevant scenario: the case of congruent AO+MI, which was also studied by the above authors.

### Congruent AO+MI

Here the observer is imagining self-execution whilst observing another person performing the same type of action. In a first approximation, the combined rectangles (b) and (d) in Figure 1 correspond to this scenario, where rectangle (b) represents the simulation of the observed person's action, and rectangle (d) the simulation of one's own action. In line with our definition of MI, the latter simulation includes a “sense of effort” (James, 1890), a sense of agency, and the imagined kinesthetic sensations that would arise during one's own motor execution.

At first sight, the idea of two simulation processes that run in parallel might appear unparsimonious, but consideration of incongruent and conflicting AO+MI states (see below) will strengthen this “dual-simulation” view. Subjectively, the contrast between AO and AO+MI is striking: Whereas in typical AO, the observer can certainly engage with the observed action and, e.g., anticipate the next steps in a high jump or in a household routine, in concurrent AO+MI one's own body schema gets “switched on” and, e.g., an observed hand movement is mapped onto one's own felt hand (and in body-oriented actions such as brushing teeth, this simulation could further include imagery of the pressure of the toothbrush on the teeth). This subjective difference is presumably reflected in the stronger activations for AO+MI in a number of cortical sites found in the studies by Macuga and Frey (2012) and Nedelko et al. (2012). Further careful manipulation of imagery instructions during AO will be required to pinpoint the neural signatures of the two concurrent processes. For example, we would expect that activations in somatosensory cortex, which are consistently found during execution and AO (Keysers et al., 2010) would be substantially enhanced by related AO+MI instructions. Surprisingly, while this region has been found to be activated during MI (e.g., Porro et al., 1996; Lotze et al., 1999; Gerardin et al., 2000), the somatosensory cortex has rarely been considered a region of interest in MI studies, and, therefore, its involvement was not extensively discussed.

The above conceptualization of AO+MI poses a number of important questions. First, it is unclear at present to what extent participants might carry out standard AO instructions as AO+MI tasks. That is, to what extent do they spontaneously imagine themselves performing the observed action, whether asked to do so or not. For example, in the prominent study by Calvo-Merino et al. (2005), observers were asked to judge “how tiring” the observed dancing movements felt,—an instruction that might well invite concurrent AO+MI. Accordingly, the frequency of spontaneous concurrent AO+MI is an important and largely ignored confound in the majority of existing neuroimaging studies on AO. The elegant fMRI study by Oosterhof et al. (2012) underlines this possibility via an in-depth comparison of activations for AO during motor execution with those for AO+MI.

Second, in our overview of different simulation states in section “Action observation and motor imagery—a continuum,” we have left it open as to when the observer might hold a sense of agency. Generally we would assume agency for all forms of MI, including AO+MI. However, it is debatable whether the involvement of motor simulation processes during AO *per se* necessarily implies the sense of agency that is so typical of MI. As pointed out above, Schubotz (2007; see also Bubic et al., 2010) has argued that predictive operations of the motor system are not limited to human actions but include a variety of inanimate events. To give a recent example, Press et al. (2012) provided evidence for responses of the ventral premotor cortex, a classical “mirror” area, in coding geometric shapes. Thus, there are certainly examples of motor cortical involvement without experienced agency. Also for observation of human action, it is conceivable that motor simulation can occur without a sense of agency and without a mapping of the observed action onto the observer's own body schema. One possibility is that the observer only holds a sense of agency when he or she co-represents the observed action as their own action via MI. If this view can be substantiated, then the notion of “understanding actions from the inside” (e.g., Rizzolatti and Sinigaglia, 2010; Gallese et al., 2011) would appear to unnecessarily conflate “default mode” motor simulation processes during AO and the sense of agency that is experienced during MI and AO+MI. In other words, we suggest that the former processes do not imply agency, and that agency typically results from co-representation sensu AO+MI. The subjective experience of AO is not the same as that of AO+MI, and, despite the activation overlap documented so far, we would predict agency-related differences in the underlying neurocognitive processes.

A third interesting question arises regarding the nature of the interactions and temporal coupling between the observed action and the two proposed simulation processes. According to the single cell recording work on mirror neurons (Rizzolatti and Sinigaglia, 2010), the temporal coupling between the observed action and its internal motor representation is tight (see also Borroni et al., 2005). Indeed, these studies indicate minimal delays between the external event and its motor representation. In contrast, at present we have no information about the possible coupling between the observed action and the MI-related simulation, or about that between both internal simulation processes.

Fourth, it is entirely possible that engaging in MI concurrently with AO draws on resources that are normally used for simulation of the observed action. For example, performance in prediction tasks might be compromised, or perhaps even enhanced, by concurrent AO+MI instructions relative to AO instructions. Competition between these two simulation processes is even more likely in the following two scenarios.

### Coordinative AO+MI

Why should an observer imagine performing action A whilst observing a different action B? When the two actions have nothing in common, this is likely going to be difficult (see section “Conflicting AO+MI”). However, one could well argue that, in overt everyday interactions, performing one action whilst seeing another action done is even more common than imitative behavior. The former is currently studied under the heading of “joint action” (Bekkering et al., 2009), where one actor responds to an observed action with a different, self-performed action, normally in pursuit of a joint or competitive goal (see our example from combat sports in the Introduction). On closer inspection, also congruent actions almost always involve a certain degree of mismatch between observed and imagined action, for example regarding the plane of motion or perspective, or both. A further, prime example of joint action is ensemble music, where the very different actions of, e.g., a jazz singer and bass player are tightly coordinated in time (see Konvalinka et al., 2010). We would then argue that the capability to engage in incongruent AO+MI, where the two actions merit coordination in one way or another, is grounded in our capacity for joint action.

Compared to joint action, imagery in AO+MI widens the scope of possible scenarios considerably. During both congruent and coordinative AO+MI, observers normally focus their MI on selected aspects of the observed action. Indeed, the idea that all degrees of freedom of a complex observed movement could be mapped in a 1:1 fashion onto the observer's motor representation is plain nonsense from the point of view of sensory anatomy alone (Vogt, 2002). Rather, in the motor simulation of an observed action, the case of a purely sensorily driven simulation (rectangle b in Figure 1) is probably quite rare and limited to movements with very few degrees of freedom, such as isolated finger movements. In the majority of cases, however, AO will be focused on certain aspects of the observed action. Already in congruent AO+MI, it is clear that MI allows for a very narrow attentional focus, for example, on the left knee joint of an observed downhill skier and on the observer's corresponding joint. Finally, coordinative AO+MI is even more flexible. Returning to our jazz ensemble, the bassist might imagine tapping along with the singer in order to fully capture her intonation and timing. Or in our example from combat sports, the observer might visually focus on the opponent's right arm whilst, in different repeats of the video, focusing on different own body parts and on their optimal imagined response. In short, coordinative AO+MI is most likely a common everyday activity, and in formal training settings in sport or motor rehabilitation, it has an abundant range of applications.

### Conflicting AO+MI

It is difficult to consider two actions, one observed and one imagined, that cannot be coordinated in some way but are solely conflicting. One example might be a skier observing a movie (showing either himself or someone else) of a slalom but simultaneously imagine himself falling during the same course, but this example might also be classified as a variant of coordinative AO+MI. In addition, and besides such (interesting) examples, it may be difficult to imagine a case of conflicting AO+MI which can be practically beneficial. However, the co-representation of conflicting instructions, task sets, or motor plans is of course a common research topic in psychology and neuroscience. For instance, most of the available research on automatic imitation effects (Heyes, 2011) relies on the contrast between compatible and incompatible visual stimuli during action planning as a methodological tool. We would thus like to illustrate possibilities for studying conflicting AO+MI, together with the other two AO+MI states, by means of an experimental paradigm that was recently developed in one of our labs (Eaves et al., 2012).

The starting point for the study by Eaves et al. (2012) was the relatively scarce evidence for automatic imitation effects in movement kinematics, as compared to the ample evidence from studies using reaction times. In each trial, participants were shown the picture of a rhythmical target action (e.g., toothbrushing), followed by a movie of an irrelevant distractor action (e.g., window wiping), followed by rhythmical execution of the target action. Across trials, the distractor action was presented in subtly different tempi, which produced a significant imitation bias during execution. In addition, the imitation bias was significantly stronger for congruent than for incongruent actions (where congruency could be regarding the type of action and/or the plane of motion). We interpreted these results in the context of Cisekand Kalaska's (2010) biased competition framework, where intended and observed actions can be represented as competing sensorimotor streams. For incongruent actions, we proposed that the competition between the two streams was strongly biased toward the intended action, and that, consequently, the coupling between the two streams was relatively weak.

A straightforward means of studying the three AO+MI states as proposed here would be to manipulate MI instructions during AO in the above paradigm. In a congruent AO+MI condition, participants could be asked to imagine performing the instructed action in synchrony with observing a congruent distractor action. Based on the results of the neuroimaging studies reviewed in section “A case for motor imagery during action observation,” we would predict an enhanced imitation bias for this condition, relative to pure distractor observation as studied in Eaves et al. (2012). A coordinative AO+MI condition could be implemented by requiring participants to imagine the instructed action in synchrony with a distractor action that is incongruent in terms of action type or plane of motion. Whilst the studies by Hove et al. (2010) and Eaves et al. (2012) indicate stronger synchronization effects for congruent actions, it is also conceivable that explicit instructions to coordinate two different actions, as envisaged here, might produce a similarly strong imitation bias for such coordinative AO+MI as for congruent AO+MI. Finally, conflicting AO+MI conditions could be studied by asking participants to imagine holding a static posture of the instructed action during AO. Here we would expect that the imitation bias would be largely abolished. A second means of studying conflicting AO+MI would be to display a static image whilst participants imagine rhythmical performance of the instructed action. Such manipulations are suitable for exploring the relative strength of the biasing effects of AO and MI. Overall, we hope that this example has illustrated that the three AO+MI states, as proposed here, can indeed be subjected to detailed experimental investigation.

### Perspective matters

As pointed out in Footnote 1, so far we have focused on third-person AO and first-person MI. Whilst a full discussion of all possible scenarios in the related 2 × 2 matrix would clearly exceed the scope of the present paper, here we briefly consider possible manipulations of visual perspective for AO only. In congruent AO+MI, observers can not only be presented with views of another person (third-person AO), but also with first-person displays that show the observer's limb(s) from a similar viewpoint as during execution. As described in section “A case for motor imagery during action observation,” Macuga and Frey (2012) had manipulated viewpoint during AO+MI but these authors only obtained negligible differences—possibly due to the rhythmical task used. Interestingly, the recent clinical trial by Cowles et al. (2013) on AO treatment for stroke patients used a setup which approximated first-person AO, where the patients observed a model actor who was sitting next to them. Observation of a video in first-person perspective, indeed combined with MI, was also used as one of the treatment conditions in Ietswaart et al.'s (2011) study. Certainly, differences in visual perspective should not be ignored when trying to account for different outcomes of clinical trials, if only since viewpoint effects have certainly been found in behavioral studies (e.g., Vogt et al., 2003). A possible advantage of third-person visual displays during AO+MI is that the observer can keep the two representations related to AO and MI more easily distinct than two first-person representations. On the other hand, the latter might be more likely to induce a sense of ownership of the observed body parts, as shown in studies on the rubber hand illusion (Haggard and Tsakiris, 2005) and on mirror-box therapy (Altschuler et al., 1999; Kang et al., 2011). Surely more experimental studies and related clinical trials are needed before firm recommendations for presentation in first- or third person perspective, or perhaps for both, can be made.

For coordinative AO+MI, it appears unnatural to present the observed action in first-person perspective, since this would not match the typical scenario of joint action (see example in the Introduction). We would thus see first-person visual presentations in coordinative AO+MI to be of greater interest for experimental studies than for clinical or training applications. The same is possibly true for first-person presentations in conflicting AO+MI. For example, would the interference effects between the first-person MI and the conflicting visual displays as predicted in section “Conflicting AO+MI” be stronger for first- or third-person presentation of the distractor movies?

## Concluding remarks

The present paper marks the return of one of us (Stefan Vogt) to issues of MI after almost two decades abstaining from the topic, which has developed so healthily in the meantime. It is true that the field of AO *per se*, which has grown with at least the same rate over this period, offers ample opportunities to study perception-action relationships, and that MI is not a mandatory step to mediate perception and action (Vogt, 1995, 1996). Furthermore, it is likely to be more attractive for an experimentalist to manipulate visual displays instead of imagery instructions, which are always open to subjective interpretation (Holmes and Calmels, 2008). However, so are visual displays! We hope to have reminded researchers in the fields of AO and MI that the two processes do not only share, at least in part, the same neural substrate (although a meta-analysis of the now available evidence from both areas of research is currently lacking), but more importantly, that they are easily carried out simultaneously, most likely not only in the laboratory but also in everyday life. As we have described in section “Multiple roles of motor imagery during action observation,” spontaneously performed AO+MI is an important and largely ignored confound in many related behavioral and neuroimaging studies. The act of “putting yourself into another person's shoes,” or “action understanding from within” (Rizzolatti and Sinigaglia, 2010) might often involve processes of MI, albeit not necessarily in the sense of a deliberate conscious effort. With this we do not wish to question the possible contribution of motor processes to action understanding and action prediction in general. Rather, we wish to distinguish the latter from a more specific AO+MI state where the observer “switches on” his or her own body schema and actively seeks to align this with the observed action,—a process that is difficult to capture without reference to the concept of MI. We have described three subtypes of concurrent AO+MI, namely congruent, coordinative, and conflicting AO+MI, where particularly the first two bear the potential for a wide range of applications in sports, occupational training as well as neurorehabilitation. AO and MI are most likely highly intertwined processes, and their joint consideration is fruitful in theoretical and applied contexts alike.

### Conflict of interest statement

The authors declare that the research was conducted in the absence of any commercial or financial relationships that could be construed as a potential conflict of interest.

## Footnotes

^1^Note that in Figure 1, unlike in Shepard's (1984) original schematic, we have deliberately conflated the transition from perception (a–c) to imagery (d–f) with a change of the observer's viewpoint: for perception, we assume observation of a third person's action (the most typical situation in which mirror neurons have been studied), and for imagery, we assume MI of one's own action (first-person perspective). Already Jeannerod (1994) pointed to an intermediate simulation state, namely dynamic visual imagery of a third person's action, which comes closest to visual perception of another person's action. For simplicity of exposition we will largely neglect this case of external visual imagery in the present paper. However, we will consider perspective manipulations in AO in section “Perspective matters.”

^2^There are substantial critiques of the idea of scientific communities and of citation analyses as sufficient basis for establishing them (Woolgar, 1976; Edge, 1979; Knorr-Cetina, 1982; Zuckerman, 1987). Our aim is not to make a bold claim for the existence of separate (a problematic term) communities (another problematic term) but more modestly to highlight how the theories and findings have not been cross-referenced in a way we see as productive.

## References

[B1] Altschuler E. L. Wisdom S. B. Stone L. Foster C. Galasko D. Llewellyn D. M. (1999). Rehabilitation of hemiparesis after stroke with a mirror. Lancet 353, 2035–2036 10.1016/S0140-6736(99)00920-4 10376620

[B2] Annett J. (1996). On knowing how to do things: a theory of motor imagery. Cogn. Brain Res 3, 65–69 10.1016/0926-6410(95)00030-5 8713546

[B3] Babiloni C. Del Percio C. Rossini P. M. Marzano N. Iacoboni M. Infarinato F. (2009). Judgment of actions in experts: a high-resolution EEG study in elite athletes. Neuroimage 45, 512–521 10.1016/j.neuroimage.2008.11.035 19111623

[B4] Babiloni C. Marzano N. Infarinato F. Iacoboni M. Rizza G. Aschieri P. (2010). “Neural efficiency” of experts' brain during judgment of actions: a high-resolution EEG study in elite and amateur karate athletes. Behav. Brain Res. 207, 466–475 10.1016/j.bbr.2009.10.034 19891991

[B5] Battaglia F. Lisanby S. H. Freedberg D. (2011). Corticomotor excitability during observation and imagination of a work of art. Front. Hum. Neurosci. 5:79 10.3389/fnhum.2011.00079 21897813 PMC3159953

[B6] Bekkering H. de Bruijn E. R. A. Cuijpers R. H. Newman-Norlund R. van Schie H. T. Meulenbroek R. (2009). Joint action: neurocognitive mechanisms supporting human interaction. Topics Cogn. Sci. 1, 340–352 10.1111/j.1756-8765.2009.01023.x 25164937

[B7] Berends H. I. Wolkorte R. Ijzerman M. J. van Putten M. J. A. M. (2013). Differential cortical activation during observation and observation-and-imagination. Exp. Brain Res. 229, 337–345 10.1007/s00221-013-3571-8 23771606

[B8] Bianco G. Feurra M. Fadiga L. Rossi A. Rossi S. (2012). Bi-hemispheric effects on corticospinal excitability induced by repeated sessions of imagery versus observation of actions. Restor. Neurol. Neurosci. 30, 481–489 10.3233/RNN-2012-120241 22850360

[B9] Borroni P. Montagna M. Cerri G. Baldissera F. (2005). Cyclic time course of motor excitability modulation during the observation of a cyclic hand movement. Brain Res. 1065, 115–124 10.1016/j.brainres.2005.10.034 16297887

[B10] Brass M. Bekkering H. Wohlschläger A. Prinz W. (2000). Compatibility between observed and executed finger movements: comparing symbolic, spatial, and imitative cues. Brain Cogn. 44, 124–143 10.1006/brcg.2000.1225 11041986

[B11] Braun S. Kleynen M. van Heel T. Kruithof N. Wade D. Beurskens A. (2013). The effects of mental practice in neurological rehabilitation; a systematic review and meta-analysis. Front. Hum. Neurosci. 7:390 10.3389/fnhum.2013.00390 23935572 PMC3731552

[B12] Bubic A. Von Cramon D. Y. Schubotz R. I. (2010). Prediction, cognition and the brain. Front. Hum. Neurosci. 4:25 10.3389/fnhum.2010.00025 20631856 PMC2904053

[B13] Buccino G. Lui F. Canessa N. Patteri H. Lagravinese G. Benuzzi (2004). Neural circuits involved in the recognition of actions performed by nonconspecifics: an fMRI study. J. Cogn. Neurosci. 16, 114–126 10.1162/089892904322755601 15006041

[B14] Calvo-Merino B. Glaser D. E. Grèzes J. Passingham R. E. Haggard P. (2005). Action observation and acquired motor skills: an fMRI study with expert dancers. Cereb. Cortex 15, 1243–1249 10.1093/cercor/bhi007 15616133

[B15] Caspers S. Zilles K. Laird A. R. Eickhoff S. B. (2010). ALE meta-analysis of action observation and imitation in the human brain. Neuroimage 50, 1148–1167 10.1016/j.neuroimage.2009.12.112 20056149 PMC4981639

[B16] Celnik P. Webster B. Glasser D. M. Cohen L. G. (2008). Effects of action observation on physical training after stroke. Stroke 39, 1814–1820 10.1161/STROKEAHA.107.508184 18403746 PMC3638075

[B17] Cisek P. Kalaska J. F. (2010). Neural mechanisms for interacting with a world full of action choices. Annu. Rev. Neurosci. 33, 269–298 10.1146/annurev.neuro.051508.135409 20345247

[B18] Clark S. Tremblay F. Ste-Marie D. (2004). Differential modulation of corticospinal excitability during observation, mental imagery and imitation of hand actions. Neuropsychologia 42, 105–112 10.1016/S0028-3932(03)00144-1 14615080

[B19] Collet C. Di Rienzo F. Hoyek N. Guillot A. (2013). Autonomic nervous system correlates in movement observation and imagery. Front. Hum. Neurosci. 7:415 10.3389/fnhum.2013.00415 23908623 PMC3726866

[B20] Collet C. Guillot A. Lebon F. MacIntyre T. Moran A. (2011). Measuring motor imagery using psychometric, behavioural, and psychophysiological tools. Exerc. Sport Sci. Rev. 39, 85–92 10.1097/JES.0b013e31820ac5e0 21206282

[B21] Conson M. Sarà M. Pistoia F. Trojano L. (2009). Action observation improves motor imagery: specific interactions between simulative processes. Exp. Brain Res. 199, 71–81 10.1007/s00221-009-1974-3 19690843

[B22] Cowles T. Clark A. Mares K. Peryer G. Stuck R. Pomeroy V. (2013). Observation-to-imitate plus practice could add little to physical therapy benefits within 31 days of stroke: translational randomized controlled trial. Neurorehabil. Neural Repair 27, 173–182 10.1177/1545968312452470 22798151

[B23] Crosbie J. H. McDonough S. M. Gilmore D. H. Wiggam M. I. (2004). The adjunctive role of mental practice in the rehabilitation of the upper limb after hemiplegic stroke: a pilot study. Clin. Rehabil. 18, 60–68 10.1191/0269215504cr702oa 14763720

[B24] Cross E. S. Kraemer D. J. M. Hamilton A. F. Kelley W. M. Grafton S. T. (2009). Sensitivity of the action observation network to physical and observational learning. Cereb. Cortex 19, 315–326 10.1093/cercor/bhn083 18515297 PMC2638791

[B25] Decety J. Jeannerod M. Germain M. Pastene J. (1991), Vegetative response during imagined movement is proportional to mental effort. Behav. Brain Res. 42, 1–5 10.1016/S0166-4328(05)80033-6 2029340

[B26] Decety J. Jeannerod M. Prablanc C. (1989). The timing of mentally represented actions. Behav. Brain Res. 34, 35–42 10.1016/S0166-4328(89)80088-9 2765170

[B27] Dijkerman H. C. Ietswaart M. Johnston M. MacWalter R. S. (2004). Does motor imagery training improve hand function in chronic stroke patients. A pilot study. Clin. Rehabil. 18, 538–549 10.1191/0269215504cr769oa 15293488

[B28] Di Pellegrino G. Fadiga L. Fogassi L. Gallese V. Rizzolatti G. (1992). Understanding motor events: a neurophysiological study. Exp. Brain Res. 91, 176–180 10.1007/BF00230027 1301372

[B29] Driskell J. E. Copper C. Moran A. (1994). Does mental practice enhance performance. J. Appl. Psychol. 79, 481–492 10.1037/0021-9010.79.4.481

[B30] Eaves D. L. Turgeon M. Vogt S. (2012). Automatic imitation in rhythmical actions: kinematic fidelity and the effects of compatibility, delay, and visual monitoring. PLoS ONE 7:e46728 10.1371/journal.pone.0046728 23071623 PMC3465264

[B31] Edge D. (1979). Quantitative measures of communication in science: a critical review. Hist. Sci. 17, 102–134 11610633 10.1177/007327537901700202

[B32] Ehrsson H. H. Geyer S. Naito E. (2003). Imagery of voluntary movement of fingers, toes, and tongue activates corresponding body-part-specific motor representations. J. Neurophysiol. 90, 3304–3316 10.1152/jn.01113.2002 14615433

[B33] Ertelt D. Hemmelmann C. Dettmers C. Ziegler A. Binkofski F. (2012). Observation and execution of upper-limb movements as a tool for rehabilitation of motor deficits in paretic stroke patients: protocol of a randomized clinical trial. BMC Neurol. 12:42 10.1186/1471-2377-12-42 22708612 PMC3495666

[B34] Ertelt D. Small S. Solodkin A. Dettmers C. McNamara A. Binkofski F. (2007). Action observation has a positive impact on rehabilitation of motor deficits after stroke. Neuroimage 36, T164–T173 10.1016/j.neuroimage.2007.03.043 17499164

[B35] Eskenazi T. Grosjean M. Humphreys G. W. Knoblich G. (2009). The role of motor simulation in action perception: a neuropsychological case study. Psychol. Res. 73, 477–485 10.1007/s00426-009-0231-5 19350271 PMC2694935

[B36] Ewan L. M. Kinmond K. Holmes P. S. (2010). An observation-based intervention for stroke rehabilitation: experiences of eight individuals affected by stroke. Disabil. Rehabil. 32, 2097–2106 10.3109/09638288.2010.481345 20455707

[B37] Feltz D. L. Landers D. M. (1983). The effects of mental practice on motor skill learning and performance: a meta-analysis. J. Psychol. 5, 25–57

[B38] Feurra M. Bianco G. Polizzotto N. R. Innocenti I. Rossi A. Rossi S. (2011). Cortico-cortical connectivity between right parietal and bilateral primary motor cortices during imagined and observed actions: a combined TMS/tDCS study. Front. Neural Circuits 5:10 10.1186/1471-2377-12-42 21909322 PMC3163809

[B39] Filimon F. Nelson J. D. Hagler D. J. Sereno M. I. (2007). Human cortical representations for reaching: mirror neurons for execution, observation, and imagery. Neuroimage 37, 1315–1328 10.1016/j.neuroimage.2007.06.008 17689268 PMC2045689

[B40] Franceschini M. Agosti M. Cantagallo A. Sale P. Mancuso M. Buccino G. (2010). Mirror neurons: action observation treatment as a tool in stroke rehabilitation. Eur. J. Phys. Rehabil. Med. 46, 517–523 20414184

[B41] Frith C. D. (2010). What is consciousness for? Pragm. Cogn. 18, 497–551 10.1075/pc.18.3.03fri

[B42] Frith C. D. (2013). The psychology of volition. Exp. Brain Res. 229, 289–299 10.1007/s00221-013-3407-6 23354664 PMC3745827

[B43] Frith C. D. Frith U. (2006). The neural basis of mentalizing. Neuron 50, 531–534 10.1016/j.neuron.2006.05.001 16701204

[B44] Frith C. D. Frith U. (2012). Mechanisms of social cognition. Annu. Rev. Psychol. 63, 287–313 10.1146/annurev-psych-120710-100449 21838544

[B45] Gallese V. Fadiga L. Fogassi L. Rizzolatti G. (1996). Action recognition in the premotor cortex. Brain 119, 593–609 10.1093/brain/119.2.593 8800951

[B46] Gallese V. Gernsbacher M. A. Heyes C. Hickok G. Iacoboni M. (2011). Mirror neuron forum. Persp. Psychol. Sci. 6, 369–407 10.1177/1745691611413392 PMC4266473 25520744

[B47] Galton F. (1883). Inquiries into Human Faculty and its Development. London: MacMillan 10.1037/14178-000

[B48] Garrison K. A. Winstein C. J. Aziz-Zadeh L. (2010). The mirror neuron system: a neural substrate for methods in stroke rehabilitation. Neurorehabil. Neural Rep. 24, 404–412 10.1177/1545968309354536 PMC11692383 20207851

[B49] Gatti R. Tettamanti A. Gough P. M. Riboldi E. Marinoni L. Buccino G. (2013). Action observation versus motor imagery in learning a complex motor task: a short review of literature and a kinematics study. Neurosci. Lett. 540, 37–42 10.1016/j.neulet.2012.11.039 23206748

[B50] Gerardin E. Sirigu A. Lehericy S. Poline J. B. Gaymard B. Marsault C. (2000). Partially overlapping neural networks for real and imagined hand movements. Cereb. Cortex 10, 1093–1104 10.1093/cercor/10.11.1093 11053230

[B51] Grèzes J. Decety J. (2001). Functional anatomy of execution, mental simulation, observation, and verb generation of actions: a meta-analysis. Hum. Brain Mapp. 12, 1–19 10.1002/1097-0193(200101)12:1<1::AID-HBM10>3.0.CO;2-V 11198101 PMC6872039

[B52] Grosbras M. H. Beaton S. Eickhoff S. B. (2012). Brain regions involved in human movement perception: a quantitative voxel-based meta-analysis. Hum. Brain Mapp. 33, 431–454 10.1002/hbm.21222 21391275 PMC6869986

[B53] Grush R. (2004). The emulation theory of representation: motor control, imagery, and perception. Behav. Brain Sci. 27, 377–442 10.1017/S0140525X04000093 15736871

[B54] Guillot A. Collet C. (2005). Duration of mentally simulated movement: a review. J. Mot. Behav. 37, 10–20 10.3200/JMBR.37.1.10-20 15642689

[B55] Guillot A. Collet C. Nguyen V. A. Malouin F. Richards C. Doyon J. (2009). Brain activity during visual versus kinesthetic imagery: an fMRI study. Hum. Brain Mapp. 30, 2157–2172 10.1002/hbm.20658 18819106 PMC6870928

[B56] Guillot A. Di Rienzo F. Moran A. MacIntyre T. Collet C. (2012a). Imagining is not doing but involves motor commands: a review of experimental data related to motor inhibition. Front. Hum. Neurosci. 6:247 10.3389/fnhum.2012.00247 22973214 PMC3433680

[B57] Guillot A. Hoyek N. Louis M. Collet C. (2012b). Understanding the timing of motor imagery: Recent findings and future directions. Int. Rev. Sport Exerc. Psychol. 5, 3–22 10.1080/1750984X.2011.623787

[B58] Haggard P. Tsakiris M. (2005). The rubber hand illusion revisited: visuotactile integration and self-attribution. J. Exp. Psychol. Hum. Percept. Perform. 31, 80–91 10.1037/0096-1523.31.1.80 15709864

[B59] Hétu S. Gregoire M. Saimpont A. Coll M. P. Eugene F. Michon P. E. (2013). The neural network of motor imagery: an ALE meta-analysis. Neurosci. Biobehav. Rev. 37, 930–949 10.1016/j.neubiorev.2013.03.017 23583615

[B60] Hétu S. Mercier C. Eugene F. Michon P. E. Jackson P. L. (2012). Modulation of brain activity during action observation: influence of perspective, transitivity and meaningfulness. PLoS ONE 6:e24728 10.1371/journal.pone.0024728 21931832 PMC3171468

[B61] Heyes C. (2011). Automatic imitation. Psychol. Bull. 137, 463–483 10.1037/a0022288 21280938

[B62] Higuchi S. Holle H. Roberts N. Eickhoff S. B. Vogt S. (2012). Imitation and observational learning of hand actions: prefrontal involvement and connectivity. Neuroimage 59, 1668–1683 10.1016/j.neuroimage.2011.09.021 21983182

[B63] Holmes P. Calmels C. (2008). A neuroscientific review of imagery and observation use in sport. J. Mot. Behav. 40, 433–445 10.3200/JMBR.40.5.433-445 18782718

[B64] Holmes P. Calmels C. (2011). Mental practice: neuroscientific support for a new approach, in Performance Psychology: A Practitioner's Guide, eds Collins D. Button A. Richards H. (Oxford: Churchill Livingstone/Elsevier), 231–244

[B65] Hove M. J. Spivey M. J. Krumhansl C. L. (2010). Compatibility of motion facilitates visuomotor synchronization. J. Exp. Psychol. Hum. Percept. Perform. 36, 1525–1534 10.1037/a0019059 20695698

[B66] Ietswaart M. Johnston M. Dijkerman H. C. Joice S. Scott C. L. MacWalter R. S. (2011). Mental practice with motor imagery in stroke recovery: randomized controlled trial of efficacy. Brain 134, 1373–1386 10.1093/brain/awr077 21515905 PMC3097892

[B67] James W. (1890). The Principles of Psychology. New York, NY: Holt 10.1037/11059-000

[B68] Jeannerod M. (1994). The representing brain: neural correlates of motor intention and imagery. Behav. Brain Sci. 17, 187–202 10.1017/S0140525X00034026

[B69] Jeannerod M. (2001). Neural simulation of action: a unifying mechanism for motor cognition. Neuroimage 14, S103–109 10.1006/nimg.2001.0832 11373140

[B70] Jeannerod M. (2006). Motor Cognition. Oxford: Oxford University Press 10.1093/acprof:oso/9780198569657.001.0001

[B71] Johansson B. B. (2012). Multisensory stimulation in stroke rehabilitation. Front. Hum. Neurosci. 6:60 10.3389/fnhum.2012.00060 22509159 PMC3321650

[B72] Kang Y. J. Ku J. Kim H. J. Park H. K. (2011). Facilitation of corticospinal excitability according to motor imagery and mirror therapy in healthy subjects and stroke patients. Ann. Rehabil. Med. 35, 747–758 10.5535/arm.2011.35.6.747 22506202 PMC3309378

[B73] Kelly A. M. C. Garavan H. (2005). Human functional neuroimaging of brain changes associated with practice. Cereb. Cortex 15, 1089–1102 10.1093/cercor/bhi005 15616134

[B74] Keysers C. Kaas J. H. Gazzola V. (2010). Somatosensation in social perception. Nat. Rev. Neurosci. 11, 417–428 10.1038/nrn2833 20445542

[B75] Kilner J. M. (2011). More than one pathway to action understanding. Trends Cogn. Sci. 15, 352–357 10.1016/j.tics.2011.06.005 21775191 PMC3389781

[B76] Kilner J. M. Friston K. J. Frith C. D. (2007). Predictive coding: an account of the mirror neuron system. Cogn. Process. 8, 159–166 10.1007/s10339-007-0170-2 17429704 PMC2649419

[B77] Knorr-Cetina K. D. (1982). Scientific communities or transepistemic arenas of research. A critique of quasi-economic models of science. Soc. Stud. Sci. 12, 101–130 10.1177/030631282012001005

[B78] Konvalinka I. Vuust P. Roepstorff A. Frith C. D. (2010). Follow you, follow me: continuous mutual prediction and adaptation in joint tapping. Q. J. Exp. Psychol. 63, 2220–2230 10.1080/17470218.2010.497843 20694920

[B79] Kosslyn S. M. (2010). Multimodal images in the brain, in The Neurophysiological Foundations of Mental and Motor Imagery, eds Guillot A. Collet C. (New York, NY: Oxford University Press), 3–16 10.1093/acprof:oso/9780199546251.003.0001

[B80] Lejeune M. Decker C. Sanchez X. (1994). Mental rehearsal in table tennis performance. Percept. Mot. Skills 79, 627–641 10.2466/pms.1994.79.1.627 7808903

[B81] Leonard G. Tremblay F. (2007). Corticomotor facilitation associated with observation, imagery and imitation of hand actions: a comparative study in young and old adults. Exp. Brain Res. 177, 167–175 10.1007/s00221-006-0657-6 16947064

[B82] Liepert J. Neveling N. (2009). Motor excitability during imagination and observation of foot dorsiflexions. J. Neural Transm. 116, 1613–1619 10.1007/s00702-009-0287-9 19680596

[B83] Loporto M. McAllister C. Williams J. Hardwick R. Holmes P. (2011). Investigating central mechanisms underlying the effects of action observation and imagery through transcranial magnetic stimulation. J. Mot. Behav. 43, 361–373 10.1080/00222895.2011.604655 21861627

[B84] Lorey B. Naumann T. Pilgramm S. Petermann C. Bischoff M. Zentgraf K. (2013a). How equivalent are the action execution, imagery, and observation of intransitive movements. Revisiting the concept of somatotopy during action simulation. Brain Cogn. 81, 139–150 10.1016/j.bandc.2012.09.011 23207575

[B85] Lorey B. Naumann T. Pilgramm S. Petermann C. Bischoff M. Zentgraf K. (2013b). Neural simulation of actions: effector- versus action-specific motor maps within the human premotor and posterior parietal area. Hum. Brain Mapp. [Epub ahead of print]. 10.1002/hbm.22246 23427116 PMC6869544

[B86] Lotze M. Montoya P. Erb M. Hulsmann E. Flor H. Klose U. (1999). Activation of cortical and cerebellar motor areas during executed and imagined hand movements: an fMRI study. J. Cogn. Neurosci. 11, 491–501 10.1162/089892999563553 10511638

[B87] Macuga K. L. Frey S. H. (2012). Neural representations involved in observed, imagined, and imitated actions are dissociable and hierarchically organized. Neuroimage 59, 2798–2807 10.1016/j.neuroimage.2011.09.083 22005592 PMC3254825

[B88] Malouin F. Jackson P. L. Richards C. L. (2013). Towards the integration of mental practice in rehabilitation programs. A critical review. Front. Hum. Neurosci. 7:576 10.3389/fnhum.2013.00576 24065903 PMC3776942

[B89] McCormick S. A. Causer J. Holmes P. S. (2012). Eye gaze metrics reflect a shared motor representation for action observation and movement imagery. Brain Cogn. 80, 83–88 10.1016/j.bandc.2012.04.010 22647575

[B90] Milner A. D. Goodale M. A. (2008). Two visual systems re-viewed. Neuropsychologia 46, 774–785 10.1016/j.neuropsychologia.2007.10.005 18037456

[B91] Moran A. (2002). In the mind's eye. The Psychologist 15, 414–415 15831399

[B92] Moran A. Guillot A. Macintyre T. Collet C. (2012). Re-imagining motor imagery: building bridges between cognitive neuroscience and sport psychology. Br. J. Psychol. 103, 224–247 10.1111/j.2044-8295.2011.02068.x 22506748

[B93] Morris T. Spittle M. Watt A. P. (2005). Imagery in Sport. Champaign, IL: Human Kinetics

[B94] Mulder T. (2007). Motor imagery and action observation: cognitive tools for rehabilitation. J. Neural Transm. 114, 1265–1278 10.1007/s00702-007-0763-z 17579805 PMC2797860

[B95] Munzert J. Lorey B. Zentgraf K. (2009). Cognitive motor processes: the role of motor imagery in the study of motor representations. Brain Res. Rev. 60, 306–326 10.1016/j.brainresrev.2008.12.024 19167426

[B96] Nedelko V. Hassa T. Hamzei F. Schoenfeld M. A. Dettmers C. (2012). Action imagery combined with action observation activates more corticomotor regions than action observation alone. J. Neurol Phys. Ther. 36, 182–188 10.1097/NPT.0b013e318272cad1 23095902

[B97] Neuper C. Scherer R. Wriessnegger S. Pfurtscheller G. (2009). Motor imagery and action observation: modulation of sensorimotor brain rhythms during mental control of a brain-computer interface. Clin. Neurophysiol. 120, 239–247 10.1016/j.clinph.2008.11.015 19121977

[B98] Nyberg L. Eriksson J. Larsson A. Marklund P. (2006). Learning by doing versus learning by thinking: an fMRI study of motor and mental training. Neuropsychologia 44, 711–717 10.1016/j.neuropsychologia.2005.08.006 16214184

[B99] Oosterhof N. N. Tipper S. P. Downing P. E. (2012). Visuo-motor imagery of specific manual actions: a multi-variate pattern analysis fMRI study. NeuroImage 63, 262–271 10.1016/j.neuroimage.2012.06.045 22766163

[B100] Page S. J. Levine P. Leonard A. (2007). Mental practice in chronic stroke: results of a randomized, placebo-controlled trial. Stroke 38, 1293–1297 10.1161/01.STR.0000260205.67348.2b 17332444

[B101] Pezzulo G. Candidi M. Dindo H. Barca L. (2013). Action simulation in the human brain: twelve questions. New Ideas Psychol. 31, 270–290 10.1016/j.newideapsych.2013.01.004

[B102] Porro C. A. Francescato M. P. Cettolo V. Diamond M. E. Baraldi P. Zuiani C. (1996). Primary motor and sensory cortex activation during motor performance and motor imagery: a functional magnetic resonance imaging study. J. Neurosci. 16, 7688–7698 8922425 10.1523/JNEUROSCI.16-23-07688.1996 PMC6579073

[B103] Press C. Catmur C. Cook R. Widmann H. Heyes C. Bird G. (2012). fMRI evidence of ‘mirror’ responses to geometric shapes. PLoS ONE 7:12 10.1371/journal.pone.0051934 23251653 PMC3522615

[B104] Ramsey R. Cumming J. Eastough D. Edwards M. G. (2010). Incongruent imagery interferes with action initiation. Brain Cogn. 74, 249–254 10.1016/j.bandc.2010.08.005 20846772

[B105] Rizzolatti G. (2005). The mirror neuron system and its function in humans. Anat. Embryol. 210, 419–421 10.1007/s00429-005-0039-z 16222545

[B106] Rizzolatti G. Fabbri-Destro M. (2010). Mirror neurons: from discovery to autism. Exp. Brain Res. 200, 223–237 10.1007/s00221-009-2002-3 19760408

[B107] Rizzolatti G. Fadiga L. Fogassi L. Gallese V. (1996). Premotor cortex and the recognition of motor actions. Cogn. Brain Res. 3, 131–141 10.1016/0926-6410(95)00038-0 8713554

[B108] Rizzolatti G. Fadiga L. Fogassi L. Gallese V. (2002). From mirror neurons to imitation: facts and speculations, in The Imitative mind: Development, Evolution, and Brain Bases, eds Meltzoff A. N. Prinz W. (Cambridge: Cambridge University Press), 247–266 10.1017/CBO9780511489969.015

[B109] Rizzolatti G. Sinigaglia C. (2010). The functional role of the parieto-frontal mirror circuit: interpretations and misinterpretations. Nat. Rev. Neurosci. 11, 264–274 10.1038/nrn2805 20216547

[B110] Roure R. Collet C. Deschaumes-Molinaro C. Delhomme G. Dittmar A. Vernet-Maury E. (1999). Imagery quality estimated by autonomic response is correlated to sporting performance enhancement. Physiol. Behav. 66, 63–72 10.1016/S0031-9384(99)00026-8 10222475

[B111] Sakamoto M. Muraoka T. Mizuguchi N. Kanosue K. (2009). Combining observation and imagery of an action enhances human corticospinal excitability. Neurosci. Res. 65, 23–27 10.1016/j.neures.2009.05.003 19463869

[B112] Solodkin A. Hlustik P. Chen E. E. Small S. L. (2004). Fine modulation in network activation during motor execution and motor imagery. Cereb. Cortex 14, 1246–1255 10.1093/cercor/bhh086 15166100

[B113] Schubotz R. I. (2007). Prediction of external events with our motor system: towards a new framework. Trends Cogn. Sci. 11, 211–218 10.1016/j.tics.2007.02.006 17383218

[B114] Schuster C. Hilfiker R. Amft O. Scheidhauer A. Andrews B. Butler J. A. (2011). Best practice for motor imagery: a systematic literature review on motor imagery training elements in five different disciplines. BMC Med. 9:75 10.1186/1741-7015-9-75 21682867 PMC3141540

[B115] Schwoebel J. Boronat C. B. Branch Coslett H. (2002). The man who executed “imagined” movements: evidence for dissociable components of the body schema. Brain Cogn. 50, 1–16 10.1016/S0278-2626(02)00005-2 12372347

[B116] Scott S. K. McGettigan C. Eisner F. (2009). A little more conversation, a little less action—candidate roles for the motor cortex in speech perception. Nat. Rev. Neurosci. 10, 295–302 10.1038/nrn2603 19277052 PMC4238059

[B117] Shallice T. (2004). The fractionation of supervisory control, in The Cognitive Neurosciences, 3rd Edn ed M. S. Gazzaniga (Cambridge, MA: MIT Press), 943–956

[B118] Shepard R. N. (1984). Ecological constraints on internal representation: Resonant kinematics of perceiving, imagining, thinking, and dreaming. Psychol. Rev. 91, 417–447 10.1037/0033-295X.91.4.417 6505114

[B119] Springer A. Parkinson J. Prinz W. (2013). Action simulation: time course and representational mechanisms. Front. Psychol. 4:387 10.3389/fpsyg.2013.00387 23847563 PMC3701141

[B120] Stürmer B. Aschersleben G. Prinz W. (2000). Correspondence effects with manual gestures and postures: a study of imitation. J. Exp. Psychol. Hum. Percept. Perform. 26, 1746–1759 10.1037/0096-1523.26.6.1746 11129371

[B121] Sully J. (1892). The Human Mind: a Text-Book of Psychology. London: Longmans Green 10.1037/12967-000

[B122] Titchener E. B. (1909). Imagery and Sensationalism. New York, NY: MacMillan

[B123] Tremblay F. Leonard G. Tremblay L. (2008). Corticomotor facilitation associated with observation and imagery of hand actions is impaired in Parkinson's disease. Exp. Brain Res. 185, 249–257 10.1007/s00221-007-1150-6 17926025

[B124] Tsukazaki I. Uehara K. Morishita T. Ninomiya M. Funase K. (2012). Effect of observation combined with motor imagery of a skilled hand-motor task on motor cortical excitability: difference between novice and expert. Neurosci. Lett. 518, 96–100 10.1016/j.neulet.2012.04.061 22580208

[B125] Van Overwalle F. Baetens K. (2009). Understanding others' actions and goals by mirror and mentalizing systems: a meta-analysis. Neuroimage 48, 564–584 10.1016/j.neuroimage.2009.06.009 19524046

[B126] Villiger M. Estevez N. Hepp-Reymond M.-C. Kiper D. Kollias S. S. Eng K. (2013). Enhanced activation of motor execution networks using action observation combined with imagination of lower limb movements. PLoS ONE 8:e72403 10.1371/journal.pone.0072403 24015241 PMC3756065

[B127] Vogt S. (1994). Imagery needs preparation, too (Commentary on a target article by M. Jeannerod). Behav. Brain Sci. 17, 226–227 10.1017/S0140525X00034324

[B128] Vogt S. (1995). On relations between perceiving, imagining and performing in the learning of cyclical movement sequences. Br. J. Psychol. 86, 191–216 10.1111/j.2044-8295.1995.tb02556.x 7795941

[B129] Vogt S. (1996). Imagery and perception-action mediation in imitative actions. Cogn. Brain Res. 3, 79–86 10.1016/0926-6410(95)00032-1 8713548

[B130] Vogt S. (2002). Visuomotor couplings in object-oriented and imitative actions, in The Imitative Mind: Development, Evolution, and Brain Bases, eds Meltzoff A. N. Prinz W. (Cambridge: Cambridge University Press), 206–220 10.1017/CBO9780511489969.012

[B131] Vogt S. Buccino G. Wohlschläger A. M. Canessa N. Shah N. Zilles K. (2007). Prefrontal involvement in imitation learning of hand actions: effects of practice and expertise. Neuroimage 37, 1371–1383 10.1016/j.neuroimage.2007.07.005 17698372

[B132] Vogt S. Taylor P. Hopkins B. (2003). Visuomotor priming by pictures of hand postures: perspective matters. Neuropsychologia 41, 941–951 10.1016/S0028-3932(02)00319-6 12667530

[B133] Vogt S. Thomaschke R. (2007). From visuo-motor interactions to imitation learning: behavioural and brain imaging studies. J. Sports Sci. 25, 497–517 10.1080/02640410600946779 17365538

[B134] Wehner T. Vogt S. Stadler M. (1984). Task-specific EMG-characteristics during mental training. Psychol. Res. 46, 389–401 10.1007/BF00309071 6522565

[B135] Weinberg R. S. (2008). Does imagery work. Effects on performance and mental skills. J. Im. Res. Sport Phys. Act. 3, 1–21 10.2202/1932-0191.1025

[B136] Williams J. Pearce A. J. Loporto M. Morris T. Holmes P. S. (2012). The relationship between corticospinal excitability during motor imagery and motor imagery ability. Behav. Brain Res. 226, 369–375 10.1016/j.bbr.2011.09.014 21939692

[B137] Woolgar S. W. (1976). The identification and definition of scientific collectivities, in Perspectives on the Emergence of Scientific Disciplines, eds Lemaine G. MacLeod R. Mulkay M. Weingart P. (The Hague: Mouton and Co.), 235–245 10.1515/9783110819038.233

[B138] Wuyam B. Moosavi S. H. Decety J. Adams L. Lansing R. W. Guz A. (1995). Imagination of dynamic exercise produced ventilatory responses which were more apparent in competitive sportsmen. J. Physiol. (Lond.) 482, 713–724 7738860 10.1113/jphysiol.1995.sp020554 PMC1157796

[B139] Zentgraf K. Munzert J. Bischoff M. Newman-Norlund R. D. (2011). Simulation during observation of human actions – theories, empirical studies, applications. Vision Res. 51, 827–835 10.1016/j.visres.2011.01.007 21277318

[B140] Ziessler M. Nattkemper D. Vogt S. (2012). The activation of effect codes in response preparation: new evidence from an indirect interference paradigm. Front. Psychol. 3:585 10.3389/fpsyg.2012.00585 23293623 PMC3533231

[B141] Zuckerman H. (1987). Citation analysis and the complex problem of intellectual influence. Scientometrics 12, 329–338 10.1007/BF02016675

